# Protective Effect of Proanthocyanidin against Diabetic Oxidative Stress

**DOI:** 10.1155/2012/623879

**Published:** 2011-09-07

**Authors:** Takako Yokozawa, Eun Ju Cho, Chan Hum Park, Ji Hyun Kim

**Affiliations:** ^1^Institute of Natural Medicine, University of Toyama, Toyama 930-0194, Japan; ^2^Department of Food Science and Nutrition, Pusan National University, Busan 609-735, Republic of Korea

## Abstract

We investigated the antidiabetic potential of proanthocyanidin and its oligomeric form in STZ-induced diabetic model rats and *db/db* type 2 diabetic mice. Proanthocyanidin ameliorated the diabetic condition by significant decreases of serum glucose, glycosylated protein, and serum urea nitrogen as well as decreases of urinary protein and renal-AGE in STZ-induced diabetic rats and decrease of serum glucose as well as significant decrease of glycosylated protein in *db/db* type 2 diabetic mice. The suppression of ROS generation and elevation of the GSH/GSSG ratio were also observed in the groups administered proanthocyanidin. Moreover, proanthocyanidin, especially its oligomeric form, affected the inflammatory process with the regulation of related protein expression, iNOS, COX-2 and upstream regulators, NF-*κ*B, and the I*κ*B-*α*. In addition, it had a marked effect on hyperlipidemia through lowering significant levels of triglycerides, total cholesterol, and NEFA. Moreover, expressions in the liver of SREBP-1 and SREBP-2 were downregulated by the administration of proanthocyanidins. The protective effect against hyperglycemia and hyperlipidemia in type 1 and 2 diabetic models was significantly strong in the groups administered the oligomeric rather than polymeric form. This suggests that oligomers act as a regulator in inflammatory reactions caused by oxidative stress in diabetes.

## 1. Introduction

Diabetes mellitus is a chronic metabolic disorder that continues to present a major worldwide health problem. It is characterized by hyperglycemia, an abnormal elevation of the blood glucose level, which has been associated with oxidative-stress [1]. Numerous studies demonstrated that oxygen free radicals are generated as a result of hyperglycemia and cause various complications of diabetes, such as nephropathy, retinopathy, and neuropathy [2, 3]. In diabetes, products of lipid peroxidation, advanced glycation end products (AGEs), and damaged DNA accumulate and eventually result in pathological diabetic complications [4–6]. Furthermore, the development of diabetes is closely related to inflammatory processes. In the absence of an appropriate compensatory response from the endogenous antioxidant network against glucotoxicity and lipotoxicity caused by hyperglycemia and hyperlipidemia under diabetes, oxidative stress becomes marked, leading to activation of the stress-sensitive intracellular signaling pathway [7, 8]. Accordingly, the attenuation of oxidative stress and regulation of stress-sensitive signaling pathways have been considered as ways to alleviate diabetes and diabetic complications.

**Figure 1:**
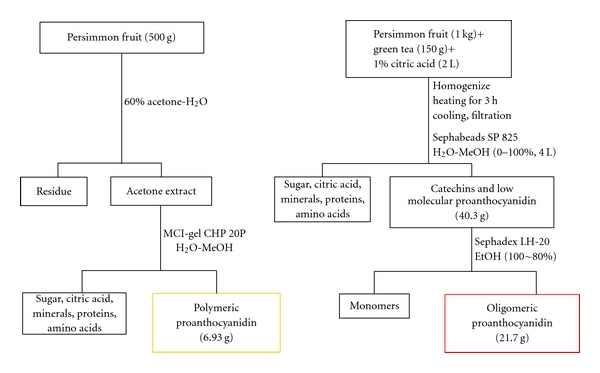
Fractionation of polymers and oligomers from proanthocyanidin of persimmon fruit.

Persimmon (*Diospyros kaki*), a deciduous fruit, is cultivated in Japan, Korea, China, Brazil, and Italy. It contains many bioactive compounds, such as polyphenols, flavonoids, terpenoids, steroids, dietary fiber, carotenoids, and minerals [9]. In particular, persimmon is a proanthocyanidin-rich food, with higher contents in the peel than in the pulp [10, 11]. Proanthocyanidin is known as condensed tannin, a member of a specific group of polyphenolic compounds, and it has been reported to exhibit powerful antioxidant activity [12, 13]. To elucidate the protective role of proanthocyanidin against diabetes in relation to its polymerization, we extracted proanthocyanidin from persimmon peel and then disintegrated polymers and oligomers from it [14], as shown in Figure 1. The degree of oligomeric polymerization was estimated as 3.3 based on quantitative HPLC analysis of thiol degradation products [15], while the unit rates of (−)-epigallocatechin, (−)-epicatechin, (−)-epigallocatechin 3-*O*-gallate, and (−)-epicatechin 3-*O*-gallate in oligomers were determined as 47, 15, 31, and 6%, respectively.

Proanthocyanidin polymers have antioxidative, antifungal, and anticancer properties [10, 16, 17]. However, the protective potential of proanthocyanidin from persimmon peel against oxidative stress and inflammation in diabetic rats has not been reported to date. Based on our previous studies [14, 18], the antidiabetic potential of proanthocyanidin and its oligomeric form *in vivo* in streptozotocin (STZ)-induced diabetic model rats and *db*/*db* type 2 diabetic mice is reviewed herein. Furthermore, the protective mechanisms against diabetes have been elucidated.

## 2. Effect of Proanthocyanidin in STZ-Induced Diabetic Rats

### 2.1. Hematological and Renal Functional Parameters

**Figure 2:**
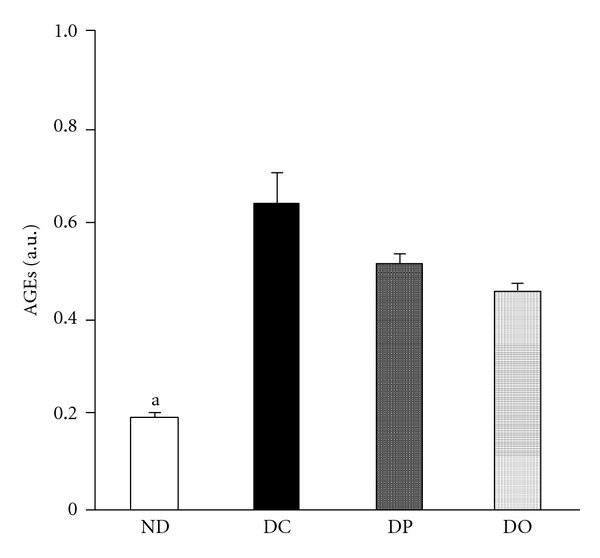
AGE levels in the kidney of STZ-induced diabetic rats. The results are expressed as the mean ± SEM. Significance: ^a^ *P* < 0.05 versus DC. ND, nondiabetic normal rats; DC, diabetic control rats; DP, polymeric proanthocyanidin 10 mg/kg body weight-treated diabetic rats; DO, oligomeric proanthocyanidin 10 mg/kg body weight-treated diabetic rats.

Under the diabetic condition, glucose itself and an increase in protein glycosylation induced by hyperglycemia are significant sources of free radicals and inducers of oxidative stress [19]. The administration of proanthocyanidin at 10 mg/kg body weight/day to STZ-induced diabetic rats led to significant decreases in the levels of glucose and glycosylated protein (Table 1). In particular, the oligomeric proanthocyanidin-administered rats showed a significant decline in these values of glucose and glycosylated protein. This indicated that proanthocyanidin attenuated the pathological condition of diabetes by controlling blood glucose and protein glycosylation. In addition, the inhibition of renal AGE formation by proanthocyanidin would be associated with attenuation of the pathogenesis of diabetic complications, since evidence that the renal accumulation of AGEs is linked to the pathogenesis of various diabetic complications has been wellestablished [20]. The levels showed a significant increase in the diabetic control group; however, the decrease in AGEs was shown in both oligomeric proanthocyanidin- and polymeric proanthocyanidin-administered rats, but only the oligomeric proanthocyanidin-administered rats showed a significant effect (Figure 2). Our study also showed that renal functional markers were improved through significant downregulation of serum urea nitrogen and decline of urinary protein level by proanthocyanidin (Table 2). The oligomeric proanthocyanidin exerted a significantly stronger protective activity than the polymeric form, suggesting that polymerization affects the functional properties of proanthocyanidin. These results are in agreement with studies showing that the maintenance of the antioxidative status plays a crucial role in protecting against renal insufficiency [21, 22].

### 2.2. Reactive Oxygen Species (ROS) Generation and Reduced Glutathione (GSH)/Oxidized Glutathione (GSSH) Redox Balance

**Figure 3:**
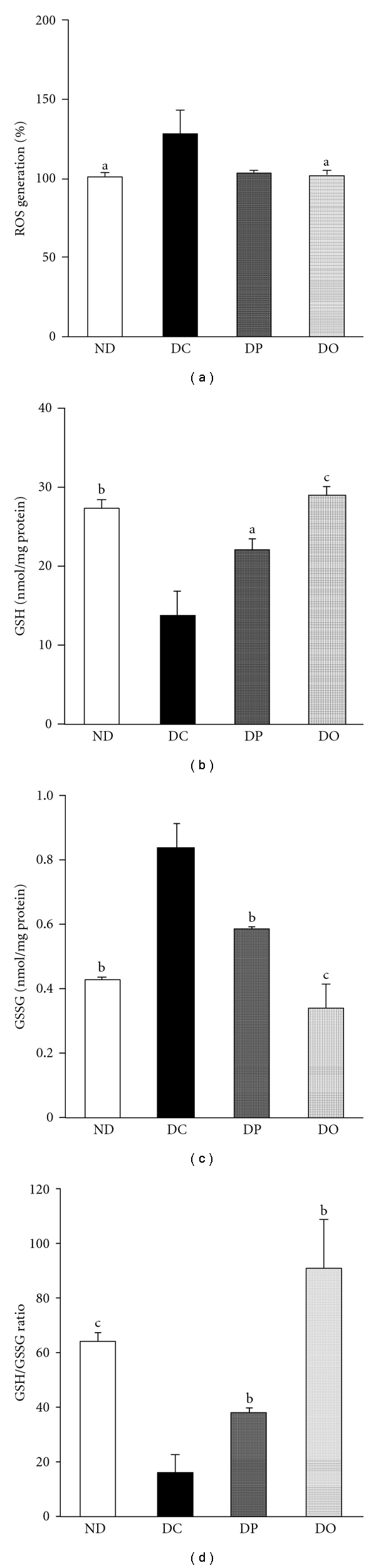
ROS generation, GSH and GSSG levels, and their ratios in the kidney of STZ-induced diabetic rats. (a) ROS generation, (b) GSH levels, (c) GSSG levels, (d) GSH/GSSG ratio. The results are expressed as the mean ± SEM. Significance: ^a^ *P* < 0.05, ^b^ *P* < 0.01, ^c^ *P* < 0.001 versus DC. ND, nondiabetic normal rats; DC, diabetic control rats; DP, polymeric proanthocyanidin 10 mg/kg body weight-treated diabetic rats; DO, oligomeric proanthocyanidin 10 mg/kg body weight-treated diabetic rats.

Oxidative stress occurs when the production of ROS overwhelms antioxidant defenses via antioxidants and antioxidative enzymes. This oxidative stress leads to cellular damage and is a causative factor in chronic degenerative diseases. In biological systems, antioxidants such as GSH, a major nonenzymatic antioxidant involved in the maintenance of the redox balance, ameliorate cellular oxidative damage. At cellular and molecular levels, redox imbalance causes the activation of redox-sensitive transcription factors that lead to inflammation [23]. Therefore, enhanced oxidative stress due to uncontrolled ROS is a major factor in both acute and chronic inflammation and inflammatory-related diseases including diabetes [24]. The effect of proanthocyanidin administration on ROS generation in the STZ-diabetic rat model is shown in Figure 3(a). The generation of ROS was significantly elevated in the diabetic control group compared with the normal group, while the administration of proanthocyanidin, both oligomers and polymers, led to a significant decrease in ROS generation. Figures 3(b)–3(d) illustrate the levels of GSH and GSSG and their ratio. The GSH level significantly decreased in the diabetic control group; however, it was significantly increased in the polymeric proanthocyanidin- and oligomeric proanthocyanidin-administered rats compared to the diabetic control group (Figure 3(b)). The GSSG level was significantly elevated in the diabetic control group, but it was significantly reduced in the polymeric proanthocyanidin- and oligomeric proanthocyanidin-administered rats (Figure 3(c)). In addition, as presented in Figure 3(d), the GSH/GSSG ratio showed a significantly marked decrease in the diabetic control group, while it increased in the polymeric proanthocyanidin- and oligomeric proanthocyanidin-administered rats, compared to the control group. The oligomer-treated rats had a higher ratio of GSH/GSSG than the polymer-treated rats through an elevation in GSH and a decline in GSSG. The increase in ROS generation under diabetes was attenuated by proanthocyanidin. This suggests that proanthocyanidin would probably ameliorate diabetic oxidative stress. Its actions are possibly related to upregulation of the GSH/GSSG ratio through an increase in GSH and decline in GSSG. The elevation of the GSH/GSSG ratio and suppression of ROS generation may be the primary role of proanthocyanidin in ameliorating oxidative stress and maintaining the redox balance. In diabetes mellitus, oxidative stress may be attributed to a combination of hyperglycemia-induced glycoxidation, sorbitol system activation, and reduced GSH synthesis due to a limited hexose monophosphate shunt. It has been reported that, under hyperglycemic conditions, as much as 30% of glucose is shunted to the polyol pathway [25], causing a marked depletion of NADPH, and, consequently, a significant decrease in the GSH level. Our current data on the GSH/GSSG ratio are in line with the results of other, which demonstrated that elevation of the GSH/GSSG ratio is effective for ameliorating oxidative stress under diabetes [26].

### 2.3. Protein Expression Related to Inflammatory Processes

**Figure 4:**
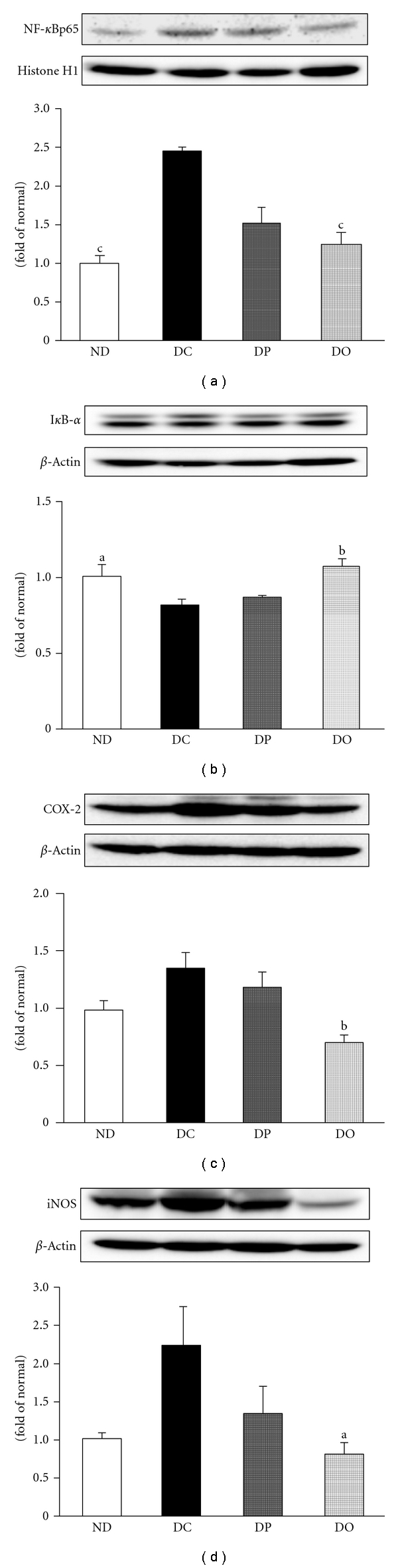
NF-*κ*B, I*κ*B-*α*, COX-2, and iNOS protein levels in the kidney of STZ-induced diabetic rats. (a) NF-*κ*B, (b) I*κ*B-*α*, (c) COX-2, (d) iNOS. The results are expressed as the mean ± SEM. Significance: ^a^ *P* < 0.05, ^b^ *P* < 0.01, ^c^ *P* < 0.001 versus DC. ND, nondiabetic normal rats; DC, diabetic control rats; DP, polymeric proanthocyanidin 10 mg/kg body weight-treated diabetic rats; DO, oligomeric proanthocyanidin 10 mg/kg body weight-treated diabetic rats.

Since redox imbalance is causally linked to inflammatory processes [27], we evaluated the expression levels of some major factors implicated in inflammation in the diabetic rat model. The inflammatory process is regulated by cyclooxygenase-2 (COX-2) and nuclear factor-*κ*B (NF-*κ*B). ROS induce the activation of NF-*κ*B activity, and NF-*κ*B, in turn, up-regulates the transcription of genes that encode enzymes such as inducible nitric oxide synthase (iNOS) and COX-2 [27, 28]. The present results showed that the protein levels of COX-2 and iNOS were increased in the diabetic oxidative rat model. As observed in Figure 4(d), the iNOS protein level in diabetic rats was increased although it did not have significant difference, but it was significantly decreased by proanthocyanidin administration; inhibition of iNOS protein expression was significantly strong in the oligomeric proanthocyanidin-administered rats than the polymeric proanthocyanidin-administered rats. The expression of COX-2 protein (Figure 4(c)) was up-regulated in the diabetic control group compared with the nondiabetic group, while the oligomeric proanthocyanidin-administered group showed significant inhibition of the expression. Feng et al. [29] reported that ROS induce the expression of COX-2 protein, the key enzyme in proinflammatory prostanoid synthesis, and COX-2 is induced readily by cytokines, hormones, growth factors, and tumor promoters in selected tissues [30, 31]. In addition, iNOS is also readily inducible by proinflammatory cytokines and has a close relationship with ROS generation. The administration of oligomeric proanthocyanidin suppressed the high-level expression of these proteins under diabetes. This suggests that modulation of COX-2 and iNOS expressions may contribute to an important protective role of proanthocyanidin against diabetes.

A molecular explanation supporting the aforementioned findings comes from the present results on NF-*κ*B expression. The NF-*κ*B complex is a heterodimer of two subunits, p50 and p65, which exist in the cytoplasm in an inactive form, and it is related to the inhibitory subunit, inhibitor protein *κ*B-*α* (I*κ*B-*α*). Molecular investigations revealed that inflammation and ROS stimulate NF-*κ*B activation by enhancing the dissociation of cytoplasmic NF-*κ*B from I*κ*B-*α*, thereby allowing NF-*κ*B to migrate to the nucleus [32], where it binds to promoters of NF-*κ*B-regulated genes to initiate gene transcription [33]. In the present study, a significant increase in the NF-*κ*Bp65 protein level and significant reduction of the I*κ*B-*α* protein level were observed in the diabetic rat (Figures 4(a) and 4(b)). However, the administration of proanthocyanidin led to a decrease in NF-*κ*Bp65 and elevation of I*κ*B-*α* protein, indicating that proanthocyanidin suppressed the translocation of NF-*κ*B to the nucleus, where it binds to the promoters of NF-*κ*B-regulated genes and initiates gene transcription. In particular, in proanthocyanidin from persimmon peel, the oligomeric form exerted stronger effects on inflammatory protein regulation than the polymeric form, suggesting the crucial role of the polymerization of proanthocyanidin in inflammation-related conditions. Our findings on NF-*κ*Bp65, COX-2, and iNOS protein levels indicate the crucial protective role of proanthocyanidin through its antidiabetic action by modulating key proinflammatory genes.

## 3. Protective Activity of Proanthocyanidin from Type 2 Diabetes with Regulation of Hyperlipidemia

### 3.1. Hematological Change and Hepatic Lipid Contents

Diabetes represents progressive and cumulative damage caused by cellular glucose and lipid metabolites. Therefore, the regulation of circulating metabolites including glucose, free fatty acids (FFA) and can be considered as a part of metabolic modulation in various tissues. In this study, we investigated glucose and glycosylated protein as hematologic factors of hyperglycemia, and triglycerides (TG), total cholesterol (TC), and nonesterified fatty acids (NEFA) as indicators of hyperlipidemia. We confirmed that *db/db* mice showed hyperglycemia as well as hyperlipidemia. As shown in Table 3, the levels of glucose and glycosylated protein in the *db/db* control group were increased significantly compared with the *m/m* group as age-matched nondiabetic misty mice. Although the glucose level was not significantly decreased by the administration of proanthocyanidins, a tendency to reduce the glucose level was exhibited by oligomers. Regarding glycosylated protein, oligomer administration led to a significant decrease in the level. Moreover, the serum concentrations of TG, TC, and NEFA were significantly elevated in the *db/db* control group compared with the *m/m* group; these concentrations were significantly reduced in the proanthocyanidin-administered groups. However, no significant difference between polymer- and oligomer-administered groups was observed. In addition, these hepatic concentrations of TG and TC were significantly decreased by proanthocyanidin administration (Table 4). In particular, the decrease in these levels was more significant in the group administered oligomers than polymers.

 From these results, the administration of proanthocyanidin ameliorated hyperglycemia through a decline in the serum level of glucose and glycosylated protein. In addition, it had a strong effect on hyperlipidemia through lowering TG, TC, and NEFA. The protective effect against hyperglycemia and hyperlipidemia was greater in the group administered the oligomeric rather than polymeric form. An increase in the level of polymerization leads to the elevation of lipase activity, leading to dietary fat digestion and absorption [34, 35]. Therefore, polymeric proanthocyanidin with high-level polymerization may suppress fat absorption in the gastrointestinal tract; consequently, the inflow of lipidemic metabolites into the blood may be inhibited. Even if lipase activity is decreased by oligomerization, oligomeric proanthocyanidin improves not only hyperlipidemia but also hepatic lipid accumulation in *db/db* mice through mechanisms distinct from those of the polymeric form.

### 3.2. Biomarkers Associated with Oxidative Stress

Hyperglycemia and elevated FFA levels result in the generation of ROS, and, consequently, increase oxidative stress. ROS not only directly damage cells by oxidizing DNA, proteins, and lipids, but also indirectly damage them by activating a variety of stress-sensitive intracellular signaling pathways such as NF-*κ*B, p38 mitogen-activated protein kinase (MAPK), NH_2_-terminal Jun kinase/stress-activated protein kinase, hexosamines, protein kinase C, AGE/receptor for AGE (RAGE), and others. Activation of these pathways results in the increased expression of numerous gene products that cause cellular damage and play a major role in the etiology of the later-stage complications of diabetes [36]. Thus, the upregulation of endogenous antioxidative systems and suppression of oxidative stress are important factors to ameliorate diabetes and its complications. As shown in Table 5, the groups administered polymers and oligomers showed a decrease in the thiobarbituric acid-reactive substance (TBARS) level, and the elevation in ROS generation was decreased by proanthocyanidin administration. The changes in TBARS and ROS generation were significantly more pronounced on the administration of oligomers rather than polymers. Our results showed that ROS generation and lipid peroxidation were increased in *db/db* mice. ROS generation induces the oxidation of membrane lipids as one of the primary events in oxidative cellular damage. Lipid peroxidation also leads to oxidant production from many molecules, and thus amplifies oxidative damage [37]. Therefore, our results suggest that *db/db* mice show increased oxidative damage due to an elevation of ROS generation induced by hyperglycemia and hyperlipidemia.

Moreover, the *db/db* control group showed significant decline of GSH/GSSG ratios through the decrease in the GSH level and increase in the GSSG level compared with the *m/m* group (Table 5). The oligomer-treated group showed elevated GSH/GSSG ratios due to the significant decrease in GSSG levels and slight increase in GSH levels, whereas the polymer-treated group did not show a significant effect. During ROS overproduction, the intracellular antioxidant GSH is oxidized to GSSG, which is then reconverted to GSH by GSH reductase. The GSH/GSSG ratio defines the so-called GSH redox state, which plays an important role in cellular activation, gene expression, mRNA stability, protein folding, metabolic regulation, and cell protection against oxidative damage [38]. Decreased GSH and increased GSSG and, consequently, down-regulation of the GSH/GSSG ratio in *db/db* mice were implicated in the disruption of the intracellular antioxidative system. In our study, the administration of oligomeric proanthocyanidin attenuated the increase in ROS generation and lipid peroxidation and elevated the GSH/GSSG ratio, whereas polymeric proanthocyanidin did not show any effect. The antioxidative effect of oligomeric proanthocyanidin is probably associated with the inhibition of glycosylated protein levels, because it is generated as glucose reacts with an amino group to form a labile Schiff base that is highly prone to oxidation and free radical generation [39]. From our data, oligomeric proanthocyanidin exhibited more effective antioxidative and antihyperlipidemic activities than polymeric proanthocyanidin. This suggests that the oligomerization of proanthocyanidin plays an important role in type 2 diabetes.

### 3.3. Regulation of Transcription Factors Related to Lipid Metabolism

**Figure 5:**
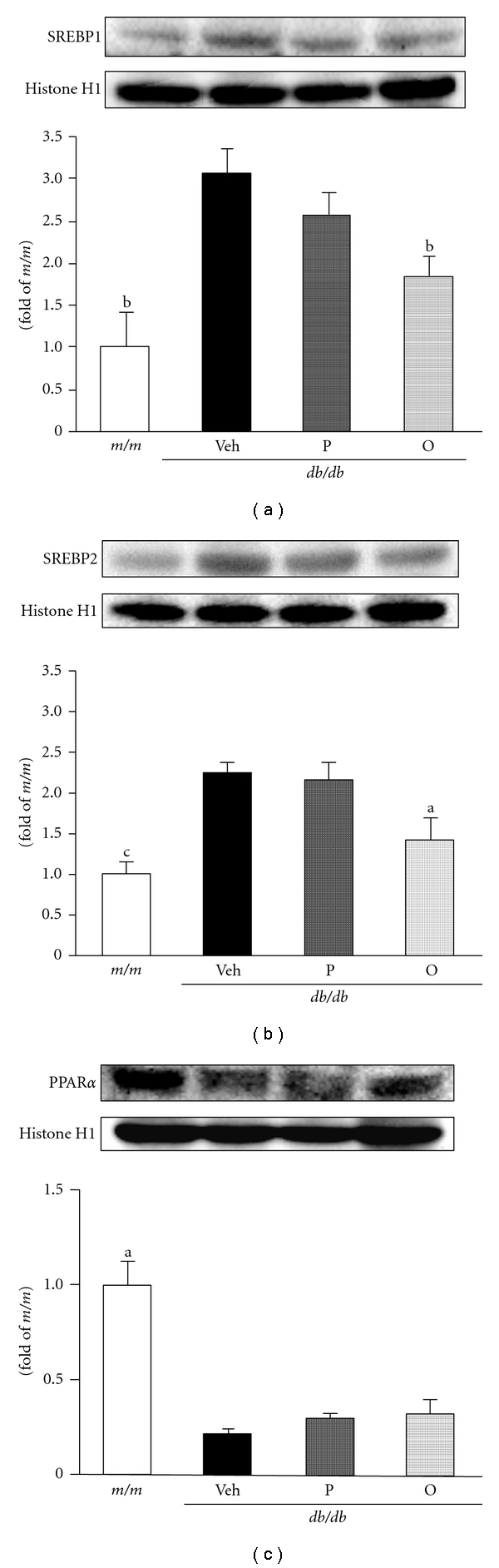
Hepatic SREBP-1, SREBP-2, and PPAR*α* protein levels in mouse model of type 2 diabetes. (a) SREBP-1, (b) SREBP-2, (c) PPAR*α*. The results are expressed as the mean ± SEM. Significance: ^a^ *P* < 0.05, ^b^ *P* < 0.01, ^c^ *P* < 0.001 versus *db/db* mice treated with vehicle. *m/m*, nondiabetic misty mice; Veh, *db/db* vehicle-treated mice; P, *db/db* mice treated with polymeric proanthocyanidin (10 mg/kg body weight); O, *db/db* mice treated with oligomeric proanthocyanidin (10 mg/kg body weight).

Lipid homeostasis is regulated by a family of membrane-bound transcription factors called sterol regulatory element binding proteins (SREBPs). SREBP-1 is a key transcription factor that nutritionally regulates the hepatic gene expression of lipogenic enzymes and TG deposition in the liver [40]. On the other hand, SREBP-2 regulates genes involved in cholesterol synthesis through the cleavage of its precursor form to an active nuclear form via interaction with SREBP cleavage activating protein and protease in a sterol-dependent manner [41]. Upregulations of SREBP-1 and SREBP-2 were reported in leptin-resistant mice such as IRS-2^−/−^ and FVB*^db/db^* mice [42, 43]. In this study, the increase in hepatic SREBP-1 and SREBP-2 in *db/db* mice was significantly down-regulated by the administration of proanthocyanidin, especially in the oligomeric form (Figure 5). This was probably related to the inhibition of hepatic TG and TC accumulations. Furthermore, peroxisome proliferator-activated receptors (PPARs), with three isoforms (*α*, *δ*, and *γ*), are also involved in the long-term regulation of lipid metabolism, and their activity is modulated by endogenous lipid-derived ligands. When PPAR*α* is activated, it promotes fatty acid oxidation, ketone body synthesis, and glucose sparing [44]. In addition, the *db/db* control group exhibited a significantly marked decrease in hepatic PPAR*α* expressions; however, administrations of both polymers and oligomers slightly increased PPAR*α* expressions (Figure 5(c)). In the current study, hepatic PPAR*α* was decreased in *db/db *mice; however, it was increased slightly by proanthocyanidin administration. In our study, we clarified that proanthocyanidin, especially in its oligomeric form, exhibits an effect on regulations of both PPAR*α* and SREBPs.

### 3.4. Modulations of Oxidative Stress-Sensitive Intracellular Signaling Pathway

**Figure 6:**
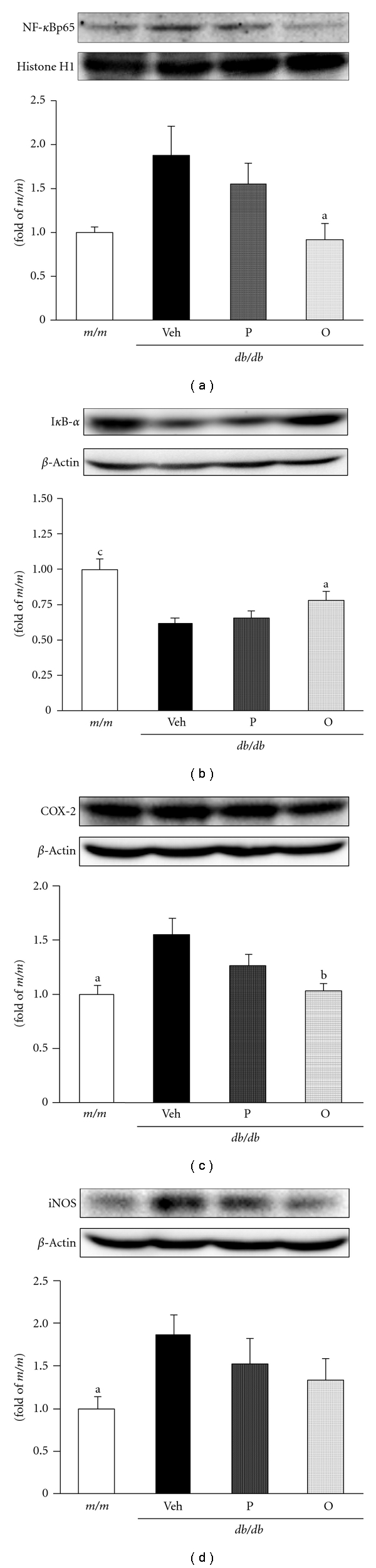
Hepatic NF-*κ*B, I*κ*B-*α*, COX-2, and iNOS protein levels in mouse model of type 2 diabetes. (a) NF-*κ*B, (b) I*κ*B-*α*, (c) COX-2, (d) iNOS. The results are expressed as the mean ± SEM. Significance: ^a^ *P* < 0.05, ^b^ *P* < 0.01, ^c^ *P* < 0.001 versus *db/db* mice treated with vehicle. *m/m*, nondiabetic misty mice; Veh, *db/db* vehicle-treated mice; P, *db/db* mice treated with polymeric proanthocyanidin (10 mg/kg body weight); O, *db/db* mice treated with oligomeric proanthocyanidin (10 mg/kg body weight).

Under type 2 diabetes, the stress-sensitive intracellular signaling pathway is altered. In particular, one major intracellular target of hyperglycemia and oxidative stress is the transcription factor NF-*κ*B. NF-*κ*B can be activated by a wide array of exogenous and endogenous stimuli including hyperglycemia, elevated FFA, ROS, tumor necrosis factor-*α*, interleukin-1*β*, other proinflammatory cytokines, AGE-binding RAGE, and p38 MAPK. The aberrant regulation of NF-*κ*B is associated with a number of chronic diseases including diabetes and atherosclerosis [45]. NF-*κ*B is present in the cytoplasm as an inactive heterodimer, consisting of p50 and p65 subunits complexed with an inhibitor protein subunit, I*κ*B. After stimulation, I*κ*B was phosphorylated by the activation of a serine kinase cascade. This event primes I*κ*B as a substrate for ubiquitination and subsequent degradation, facilitating NF-*κ*B heterodimer translocation to the nucleus. NF-*κ*B regulates the expression of a large number of genes, including growth factors, proinflammatory cytokines, and others [45, 46]. NF-*κ*B is involved in the regulation of COX-2 and iNOS expressions that mediate inflammatory processes [28]. NF-*κ*B expression in the *db/db* control group was up-regulated, while it was significantly suppressed by the administration of oligomers (Figure 6(a)). I*κ*B-*α* expression was down-regulated in the *db/db* control group, but its expression intensity was significantly increased by oligomer administration. On the other hand, the polymer-treated group showed no significant change in NF-*κ*B and I*κ*B expressions (Figures 6(a) and 6(b)). However, proanthocyanidin administration, especially oligomers rather than polymers, suppressed protein expressions of COX-2 and iNOS (Figures 6(c) and 6(d)). In *db/db* mice, hepatic NF-*κ*B was up-regulated with the down-regulation of I*κ*B compared with *m/m* mice, while the administration of proanthocyanidin, especially in its oligomeric form, led to the down-regulation of NF-*κ*B with the upregulation of I*κ*B. NF-*κ*B activation induces insulin resistance by lipid/fatty acid infusion and the inhibition of insulin signaling by lipid metabolites such as diacylglycerol and ceramide [47]. Accordingly, our results suggest that the regulation of NF-*κ*B and I*κ*B by oligomers is associated with the amelioration of hyperlipidemia and hyperglycemia. Moreover, COX-2 and iNOS mediate inflammation-mediated/induced insulin resistance. The expressions were increased in *db/db* compared with *m/m* mice. COX-2 and iNOS expressions were up-regulated under insulin resistance, obesity, hyperglycemia, and oxidative stress [48]. Their down-regulations by oligomers shown by the present results indicated the regulation of hyperlipidemia with the inhibition of TG and TC deposition. The present results suggest the crucial role of oligomers in stress-activated signaling pathways such as NF-*κ*B, COX-2, and iNOS.

## 4. Conclusion

Proanthocyanidin exerted a protective role against hyperglycemia and hyperglycemia-related changes through modulating glucose, glycosylated protein, serum urea nitrogen, urinary protein, and renal AGEs in STZ-diabetic rats. Based on the results, we propose that the suppression of oxidative stress-related inflammation is a plausible mechanism underlying the protective effect of proanthocyanidin in diabetic rats. The administration of proanthocyanidin to STZ-induced type 1 diabetic rats and *db*/*db* type 2 diabetic mice attenuated oxidative stress through the inhibition of lipid peroxidation, ROS generation, and elevation of the GSH/GSSG ratio. In addition, proanthocyanidin regulated the expression of proteins related to inflammation such as iNOS, COX-2, NF-*κ*Bp65, and I*κ*B-*α* protein. In particular, the oligomeric form of proanthocyanidins ameliorated oxidative damage and lipid deposition in the liver more effectively than the polymeric form. Oligomerization may be associated with ameliorations of oxidative stress and abnormal lipid metabolism. The oligomeric form of proanthocyanidins reversed hyperlipidemia in parallel with regulations of hepatic SREBP-1 and SREBP-2 expressions. The present study suggests that proanthocyanidins, especially oligomers, act as a regulator in inflammatory reactions caused by oxidative stress in diabetes.

## Figures and Tables

**Table 1:** Serum glucose and glycosylated protein levels in STZ-induced diabetic rats.

Group	Glucose	Glycosylated protein
	(mg/dL)	(nmol/mg protein)
ND	116.5 ± 4.2^b^	28.30 ± 1.20^b^
DC	591.3 ± 18.4	46.84 ± 1.42
DP	583.8 ± 18.0	44.11 ± 1.13
DO	532.8 ± 9.0^a^	40.60 ± 1.90^a^

The results are expressed as the mean ± SEM. Significance: ^a^ *P* < 0.05, ^b^ *P* < 0.001 versus DC. ND, nondiabetic normal rats; DC, diabetic control rats; DP, polymeric proanthocyanidin 10 mg/kg body weight-treated diabetic rats; DO, oligomeric proanthocyanidin 10 mg/kg body weight-treated diabetic rats.

**Table 2:** Renal function parameters in STZ-induced diabetic rats.

Group	Serum urea nitrogen	Urinary protein
	(mg/dL)	(mg/day)
ND	19.3 ± 0.8^b^	13.10 ± 0.76
DC	33.1 ± 1.5	23.02 ± 4.78
DP	30.3 ± 0.6	16.64 ± 2.19
DO	24.0 ± 1.3^a^	13.06 ± 0.22

The results are expressed as the mean ± SEM. Significance: ^a^ *P* < 0.01, ^b^ *P* < 0.001 versus DC. ND, nondiabetic normal rats; DC, diabetic control rats; DP, polymeric proanthocyanidin 10 mg/kg body weight-treated diabetic rats; DO, oligomeric proanthocyanidin 10 mg/kg body weight-treated diabetic rats.

**Table 3:** Hematological analyses in mouse model of type 2 diabetes.

Group	Dose (mg/kg B.W./day)	Glucose (mg/dL)	Glycosylated protein (nmol/mg protein)	TG (mg/dL)	TC (mg/dL)	NEFA (mEq/L)
*m/m *	—	127.8 ± 13.4^c^	57.3 ± 7.5^b^	68.4 ± 5.8^c^	68.8 ± 2.2^c^	0.72 ± 0.07^c^
*db/db*						
Vehicle	—	663.4 ± 58.4	112.1 ± 12.6	152.2 ± 20.2	167.5 ± 5.9	1.47 ± 0.07
Polymers	10	607.8 ± 69.7	98.6 ± 18.3	76.3 ± 9.5^b^	150.3 ± 3.9^a^	1.06 ± 0.07^c^
Oligomers	10	572.7 ± 70.5	75.5 ± 8.9^a^	75.2 ± 3.3^c^	149.8 ± 4.9^a^	1.06 ± 0.07^c^

The results are expressed as the mean ± SEM. Significance: ^a^ *P* < 0.05, ^b^ *P* < 0.01, ^c^ *P* < 0.001 versus *db/db* mice treated with vehicle.

**Table 4:** Liver weight and hepatic TG and TC contents in mouse model of type 2 diabetes.

Group	Dose (mg/kg B.W./day)	Weight (g)	TG (mg/liver/10 g B.W.)	TC (mg/liver/10 g B.W.)
*m/m*	—	1.21 ± 0.02^b^	23.5 ± 4.6^b^	32.2 ± 1.7^c^
*db/db*				
Vehicle	—	4.25 ± 0.15	78.2 ± 9.2	54.0 ± 2.5
Polymers	10	4.02 ± 0.10	59.1 ± 3.4	51.7 ± 1.7
Oligomers	10	3.98 ± 0.20	56.9 ± 2.1^a^	36.8 ± 3.6^c^

The results are expressed as the mean ± SEM. Significance: ^a^ *P* < 0.05, ^b^ *P* < 0.01, ^c^ *P* < 0.001 versus *db/db* mice treated with vehicle.

**Table 5:** Hepatic biomarkers associated with oxidative stress in mouse model of type 2 diabetes.

Group	Dose (mg/kg B.W./day)	TBARS (nmol/mg protein)	ROS generation (%)	GSH (*μ*mol/mg protein)	GSSG (*μ*mol/mg protein)	GSH/GSSG
*m/m *	—	0.63 ± 0.07^a^	100.3 ± 9.3^c^	6.63 ± 0.36^a^	4.65 ± 0.52	1.49 ± 0.12^c^
*db/db*						
Vehicle	—	1.18 ± 0.20	248.2 ± 30.3	4.91 ± 0.56	5.67 ± 0.57	0.86 ± 0.03
Polymers	10	1.11 ± 0.12	206.5 ± 15.7	4.63 ± 0.22	5.45 ± 0.24	0.85 ± 0.02
Oligomers	10	0.71 ± 0.07^a^	185.1 ± 12.3	4.07 ± 0.36	3.76 ± 0.31^a^	0.98 ± 0.05^b^

The results are expressed as the mean ± SEM. Significance: ^a^ *P* < 0.05, ^b^ *P* < 0.01, ^c^ *P* < 0.001 versus *db/db* mice treated with vehicle.
